# Hydrogen Sulfide Donor GYY4137 Rescues NRF2 Activation in Respiratory Syncytial Virus Infection

**DOI:** 10.3390/antiox11071410

**Published:** 2022-07-21

**Authors:** Aline Haas de Mello, Tianshuang Liu, Roberto P. Garofalo, Antonella Casola

**Affiliations:** 1 Department of Pediatrics, The University of Texas Medical Branch, Galveston, TX 77555, USA; alhaasde@utmb.edu (A.H.d.M.); tiliu@utmb.edu (T.L.); rpgarofa@utmb.edu (R.P.G.); 2 Department of Microbiology and Immunology, The University of Texas Medical Branch, Galveston, TX 77555, USA

**Keywords:** hydrogen sulfide, H_2_S donor, GYY4137, respiratory syncytial virus, NRF2, antioxidant enzymes

## Abstract

Respiratory syncytial virus (RSV) can cause severe respiratory illness in infants, immunocompromised, and older adults. Despite its burden, no vaccine or specific treatment is available. RSV infection is associated with increased reactive oxygen species (ROS) production, degradation of the transcription factor nuclear factor erythroid 2-related factor 2 (NRF2), and decreased antioxidant enzymes (AOEs), leading to oxidative damage and lung injury. Hydrogen sulfide (H_2_S) is an endogenous gaseous molecule that plays a physiological role in numerous cellular processes and a protective role in multiple pathological conditions, displaying vasoactive, cytoprotective, anti-inflammatory, and antioxidant activities. H_2_S can promote NRF2 activation through the sulfhydration of Kelch-like ECH-associated protein 1, the cytoplasmic repressor of NRF2. Here we investigated whether increasing cellular H_2_S levels could rescue NRF2 and NRF2-dependent gene expression in RSV-infected primary airway epithelial cells. We found that treatment with the H_2_S donor GYY4137 significantly increased NRF2 levels and AOEs gene expression by decreasing KEAP1 levels, and by modulating pathways involved in RSV-induced NRF2 degradation, such as NRF2 ubiquitination, and promyelocytic leukemia (PML) protein levels. These results suggest that the administration of exogenous H_2_S can positively impact the altered redox balance associated with RSV infection, which represents an important determinant of RSV-induced lung disease.

## 1. Introduction

Respiratory syncytial virus (RSV), a member of the *Pneumoviridae* family, is a common respiratory virus that continues to be a burden on health care and society. Premature infants, immunocompromised individuals, and older adults are at a higher risk of developing a lower respiratory tract infection (LRTI), such as bronchiolitis and pneumonia, leading to hospitalization. Currently, there is no commercially available vaccine or specific treatment, and treatment for RSV is mainly supportive [1,2].

RSV infection induces reactive oxygen species (ROS) overproduction, oxidative stress, and oxidative injury to the lungs [3,4,5,6,7,8]. The detrimental effects of ROS are usually controlled through the activation of the transcription factor nuclear factor erythroid 2-related factor 2 (NRF2), which is the master regulator of the expression of antioxidant enzymes (AOEs) [9]. Under normal conditions, Kelch-like ECH-associated protein 1 (KEAP1) binds to NRF2, retains it in the cytoplasm, and leads it to ubiquitination and proteasomal degradation. Several cysteine residues in KEAP1 serve as sensors for stress signals, and their modification causes conformational changes in KEAP1, resulting in the dissociation of the KEAP1-NRF2 complex, enabling NRF2 to translocate to the nucleus [9,10,11,12]. In the nucleus, NRF2 binds to the antioxidant response element (ARE) and activates the transcription of its target genes [11,13], such as NAD(P)H quinone oxidoreductase-1 (NQO1), superoxide dismutases (SOD1, SOD2, SOD3), catalase, glutathione peroxidases (GPx1, GPx2), glutathione S-transferases (GSTs), heme oxygenase-1 (HO-1), glutamate-cysteine ligase catalytic subunit (GCLC), and modifier subunit (GCLM) [14]. We have shown that RSV infection induces the NRF2 degradation and decreased AOEs expression in cells, mice, and children, leading to oxidative damage, an important pathogenetic component of RSV lung disease [4,5,15,16,17]. The activation of NRF2 plays an essential role in preventing injury induced by oxidative stress [18], and, therefore, represents a potential approach to ameliorate RSV-induced oxidative damage.

Hydrogen sulfide (H_2_S) is a gaseous molecule, initially viewed as a toxic gas and environmental hazard, which was found in recent years to be synthesized by mammalian cells and to participate in several biological functions, as it modulates multiple cellular signaling pathways. H_2_S is considered to be the third cellular gasotransmitter—besides nitric oxide (NO) and carbon monoxide (CO). Gasotransmitters are small molecules of endogenous gases with important physiological functions [19,20,21,22]. H_2_S has vasoactive, cytoprotective, anti-inflammatory, and antioxidant properties [21,22,23,24,25]. H_2_S can regulate the cellular antioxidant defenses by affecting the interaction between NRF2 and KEAP1 [12,26,27]. The increased H_2_S cellular levels are associated with the increased nuclear localization of NRF2 through a specific KEAP1 post-translational modification (PTM), called sulfhydration (also known as persulfidation). This process causes conformational changes in KEAP1, resulting in dissociation from NRF2. The released NRF2 translocates to the nucleus to regulate the transcription of the antioxidant defense genes [12,24,28]. The H_2_S donors, compounds that degrade in response to a specific trigger to release H_2_S, have been used to demonstrate how H_2_S administration exerts therapeutic effects in various experimental conditions [21,29]. GYY4137 is a slow-releasing H_2_S donor compound that releases H_2_S upon hydrolysis and was designed to mimic and model the slow rate of endogenous, physiological H_2_S production [21].

The redox balance between the oxidant and antioxidant species might directly or indirectly affect the progression and outcome of a viral infection [30]. Considering that RSV infection induces the NRF2 degradation and that H_2_S can activate NRF2, we investigated whether increasing cellular H_2_S levels with GYY4137 treatment could rescue NRF2 and the expression of its target genes in airway epithelial cells infected with RSV, and to explore the mechanisms involved.

## 2. Materials and Methods

### 2.1. RSV Preparation

The RSV Long strain was grown in HEp-2 cells (ATCC, Manassas, VA, USA) and purified by ultracentrifugation in sucrose density gradient. The titer of the viral pools was determined by methylcellulose plaque assay and ranged from eight to nine log_10_ plaque-forming units (PFU)/mL. The virus pools were aliquoted, quick-frozen on dry ice and alcohol, and stored at −80 °C.

### 2.2. Primary Cells Culture Conditions and RSV Infection

The primary human small airway epithelial cells (SAECs) (CC-2547, Lonza, Walkersville, MD, USA), isolated from the normal human lung tissue of cadaveric donors, were cultured according to the manufacturer’s instructions. The cells were maintained in Small Airway Epithelial Cell Growth Medium, which consists of Small Airway Epithelial Cell Basal Medium with supplements and growth factors (CC-3119 and CC-4124, Lonza, Walkersville, MD, USA). When the cells were used for RSV infection, the growth medium was changed to a basal medium lacking supplements and growth factors, 3 h before infection. The cells were kept in the basal medium throughout the length of the experiments. At 90% confluence, the cells were infected with RSV at the multiplicity of infection (MOI) of 3 and incubated for 1 h at 37 °C in 5% CO_2_, to allow viral adsorption.

### 2.3. Treatments

The slow-releasing H_2_S donor GYY4137 was purchased either from Sigma-Aldrich, St. Louis, MO, USA (SML0100) or Cayman Chemical, Ann Arbor, MI, USA (13345). A solution of 80 mM was prepared in sterile phosphate buffered saline (PBS). This solution was further diluted to a working concentration (1 to 5 mM) in Small Airway Epithelial Cell Basal Medium. The GYY4137 was added to the cells 1 h after RSV, after removal of the viral inoculum. The controls received the same volume of the vehicle. We previously tested the cytotoxicity of the GYY4137, and no significant cytotoxic effect was observed using the 5 mM dose [31]. Pal et al. [32] also reported 5 mM as a non-toxic dose of GYY4137.

### 2.4. Western Blot

In most of the experiments, the total cell lysates for Western blot were prepared with RIPA buffer (9806, Cell Signaling, Danvers, MA, USA), and the protein concentration was determined with a Pierce BCA Protein Assay Kit (23225, Thermo Fisher Scientific, Waltham, MA, USA). For the promyelocytic leukemia (PML) protein Western blot, the cells were lysed directly with SDS loading buffer (B7703S, New England BioLabs, Ipswich, MA, USA), followed by a brief sonication (10–15 s) to reduce the sample viscosity. Equal amounts (30–40 μg) of the proteins were separated by SDS-PAGE and transferred onto polyvinylidene difluoride (PVDF) membrane. Nonspecific binding was blocked by immersing the membrane in Tris-buffered saline-Tween (TBST) blocking solution containing 5% skim milk powder. After blocking, the membranes were incubated with the primary antibody overnight at 4 °C, followed by the appropriate secondary antibody for 1 h at room temperature. The proteins were detected using enhanced chemiluminescence (ECL) reagents. The densitometric analysis was performed using UVP VisionWorks LS Analysis Software. The primary antibodies used were anti-NRF2 (ab62352, Abcam, Cambridge, UK), anti-RSV (7950-0104, Bio-Rad Laboratories, Hercules, CA, USA), anti-KEAP1 (sc-365626, Santa Cruz Biotechnology, Dallas, TX, USA), and anti-PML protein (ab179466, Abcam, Cambridge, UK). β-Actin was used as the loading control (A1978, Sigma-Aldrich, St. Louis, MO, USA; sc-47778 HRP, Santa Cruz Biotechnology, Dallas, TX, USA).

### 2.5. Reverse Transcription—Quantitative Polymerase Chain Reaction (RT-qPCR)

Total RNA was extracted using RNeasy mini kit (74104, Qiagen, Hilden, Germany). On-column DNase digestion was performed using RNAse-Free Dnase Set (79254, Qiagen, Hilden, Germany). The RNA samples were quantified using a DS-11 Spectrophotometer (DeNovix, Wilmington, DE, USA). The synthesis of the cDNA was performed with 1 μg of total RNA in a 20 µL reaction, using iScript Reverse Transcription Supermix (1708841, Bio-Rad Laboratories, Hercules, CA, USA). The prepared cDNA was diluted five times with molecular biology grade water, and qPCR amplification was completed using 2 µL of cDNA in a total reaction volume of 20 µL containing primer pair and probe (forward primer, reverse primer, and probe) and TaqMan Universal Master Mix (4440040, Applied Biosystems, Waltham, MA, USA), or primer pair and Universal SYBR Green Fast qPCR (RK21203, ABclonal Technology, Woburn, MA, USA). A total of 18S rRNA was used as the housekeeping gene for normalization. The qPCR assays were run in the Bio-Rad CFX Connect Real-Time System. The Ct values were analyzed in Microsoft Excel, using the delta-delta Ct method (2^–∆∆Ct^ method). The SOD1, catalase, NQO1, GCLC, GCLM, and PML primer sequences are available upon request.

### 2.6. NRF2 Ubiquitination (Immunoprecipitation—Western Blot)

For the NRF2 ubiquitination determination, the cells were harvested with RIPA buffer (9806, Cell Signaling, Danvers, MA, USA) and the lysates were submitted to three freeze–thaw cycles. After centrifugation, the supernatants were collected for use and the protein concentration was determined with Pierce BCA Protein Assay Kit (23225, Thermo Fisher Scientific, Waltham, MA, USA). The NRF2 was immunoprecipitated from the total cell lysates (containing 1 mg of total protein) with 8 μg of anti-NRF2 antibody (PM069, MBL International Corporation, Woburn, MA, USA) and the NRF2 ubiquitination was analyzed by Western blot with anti-ubiquitin antibody (AUB01-HRP, Cytoskeleton, Denver, CO, USA). The membranes were stripped and reprobed with anti-NRF2 antibody (ab62352, Abcam, Cambridge, UK) to determine the levels of immunoprecipitated NRF2. A sample, immunoprecipitated with 8 μg of Normal Rabbit IgG (2729, Cell Signaling, Danvers, MA, USA), was used as a control for nonspecific bindings. A sample (40 μg of protein) of the pre-immunoprecipitation lysate was also analyzed by Western blot with anti-NRF2 antibody (ab62352, Abcam, Cambridge, UK) to show levels of NRF2 before immunoprecipitation (input). The densitometric analysis was completed using the UVP VisionWorks LS Analysis Software (UVP, Upland, CA, USA). The ubiquitinated NRF2 was normalized by immunoprecipitated NRF2.

### 2.7. Histone Deacetylase (HDAC) Activity

For HDAC activity measurement, the nuclear fraction was isolated from the cells as described in Schreiber et al. [33] with slight modifications. The class I and II HDAC activity was measured in nuclear extracts, using the HDAC Fluorometric Activity Assay Kit (10011563, Cayman Chemical, Ann Arbor, MI, USA) according to the manufacturer’s instructions. The HDAC activity in nmol/min/mL was normalized to protein concentration, determined with a Pierce BCA Protein Assay Kit (23225, Thermo Fisher Scientific, Waltham, MA, USA), and expressed as nmol/min/mg protein.

### 2.8. Statistical Analysis

The data were analyzed by one-way or two-way analysis of variance (ANOVA), followed by Tukey’s test using GraphPad Prism 9. The results are expressed as mean ± standard error of the mean (SEM) and *p* < 0.05 was considered statistically significant.

## 3. Results

### 3.1. H_2_S Donor GYY4137 Rescues NRF2 Levels and NRF2-Dependent Gene Expression in RSV Infection

**Figure 1:**
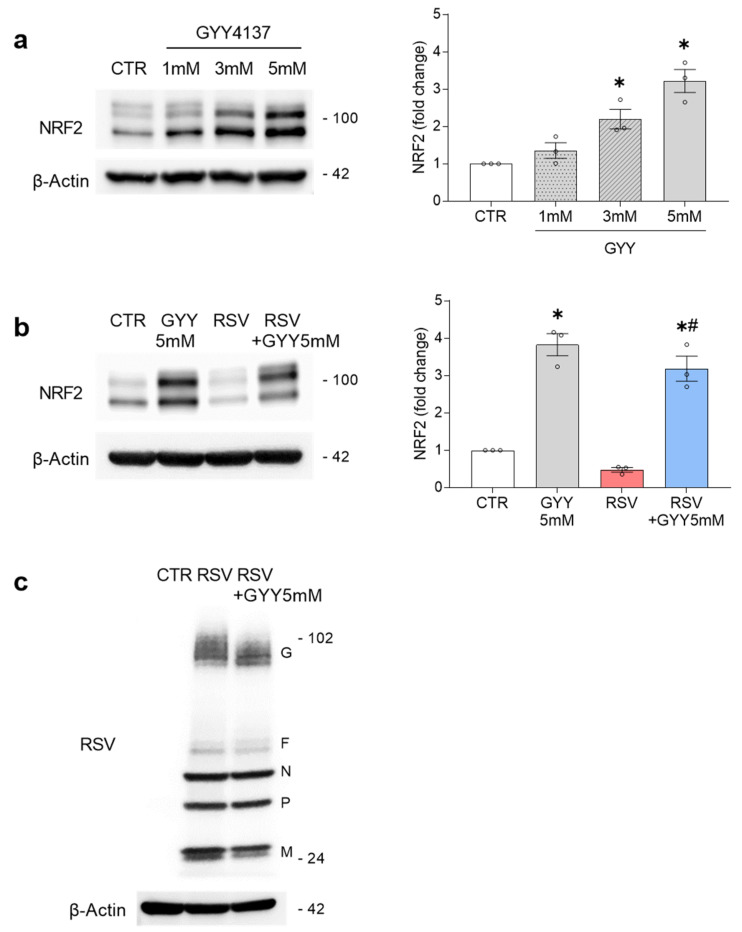
H_2_S donor GYY4137 activates and rescues NRF2 in RSV infection. (**a**) Primary human small airway epithelial cells (SAECs) were treated with 1, 3, or 5 mM GYY4137 or its vehicle. Cells were harvested 17 h after treatment and total cell lysates were analyzed by Western blot with anti-NRF2 antibody. The membrane was reprobed with anti-β-actin antibody for loading control. Western blot image is one representative of three independent experiments. The graph shows the densitometric analysis of NRF2 after normalization to β-actin expressed as mean ± SEM. Data were analyzed by one-way ANOVA followed by Tukey’s test (* *p* < 0.05 vs. CTR); (**b**) SAECs uninfected and infected with RSV were treated with 5 mM GYY4137 (or vehicle). GYY4137 was added 1 h after RSV infection. Cells were harvested 18 h post-infection (hpi) and total cell lysates were analyzed by Western blot with anti-NRF2 antibody. The membrane was reprobed with anti-β-actin antibody for loading control. Western blot image is one representative of three independent experiments. The graph shows the densitometric analysis of NRF2 after normalization to β-actin expressed as mean ± SEM. Data were analyzed by two-way ANOVA followed by Tukey’s test (* *p* < 0.05 vs. CTR; # *p* < 0.05 vs. RSV); (**c**) SAECs were infected with RSV followed by treatment with 5 mM GYY4137. Cells were harvested 18 hpi and RSV proteins were detected in total cell lysates by Western blot using anti-RSV antibody. RSV proteins corresponding bands are indicated on the right. The membrane was reprobed with anti-β-actin for loading control. Western blot image is one representative of two independent experiments.

As H_2_S has been shown to activate NRF2, we investigated if the slow-releasing H_2_S donor, GYY4137, could rescue NRF2 and the expression of its target genes in cells infected with RSV. We performed our experiments in primary human SAECs, which represent a major target of RSV infection. We initially tested the effect of different concentrations of GYY4137 (1, 3, and 5 mM) on the NRF2 activation in SAECs. The cells were harvested 17 h after treatment and the total cell lysates were analyzed by Western blot with anti-NRF2 antibody. The results showed that the GYY4137 increased the NRF2 levels in a dose-dependent manner, with 5 mM showing the highest induction (Figure 1a). To confirm that this was an effect of H_2_S, we also tested sodium hydrosulfide (NaHS), a fast-releasing H_2_S donor, which also led to increased NRF2 levels in SAECs (Figure S1, Supplementary Materials). One should note that the effects of the H_2_S donors depend not only on the concentration, but also on the rate of H_2_S generation. Rapid-release H_2_S donors, such as NaHS, are used in lower concentrations than the slow-release H_2_S donor GYY4137, which releases H_2_S at a very low level but constantly.

Then, we investigated if treatment with GYY4137 would increase the NRF2 protein levels in the context of RSV infection, where NRF2 is degraded through several mechanisms. The SAECs were infected with RSV and treated with 5 mM GYY4137 (or vehicle). The cells were harvested 18 h post-infection (hpi) and the total cell lysates were analyzed by Western blot with anti-NRF2 antibody. Our results showed that the H_2_S donor administration was able to rescue the NRF2 degradation during RSV infection, leading to NRF2 cellular levels almost comparable to the GYY-treated uninfected cells (Figure 1b).

We previously demonstrated that GYY4137 has antiviral activity against RSV, by affecting a late stage of viral replication (assembly/release), without having a significant effect on the viral gene transcription, protein synthesis, or genome replication [31,34,35]. To confirm that the GYY4137 treatment did not significantly affect the initial intracellular steps of RSV replication in SAECs, we determined the RSV protein expression in response to the GYY4137 treatment. The SAECs were infected with RSV, treated with GYY4137, and harvested 18 hpi. The RSV proteins were detected in the total cell lysates by Western blot, using an anti-RSV antibody. The results showed that the RSV proteins levels were not greatly affected by the GYY4137 treatment (Figure 1c).

**Figure 2:**
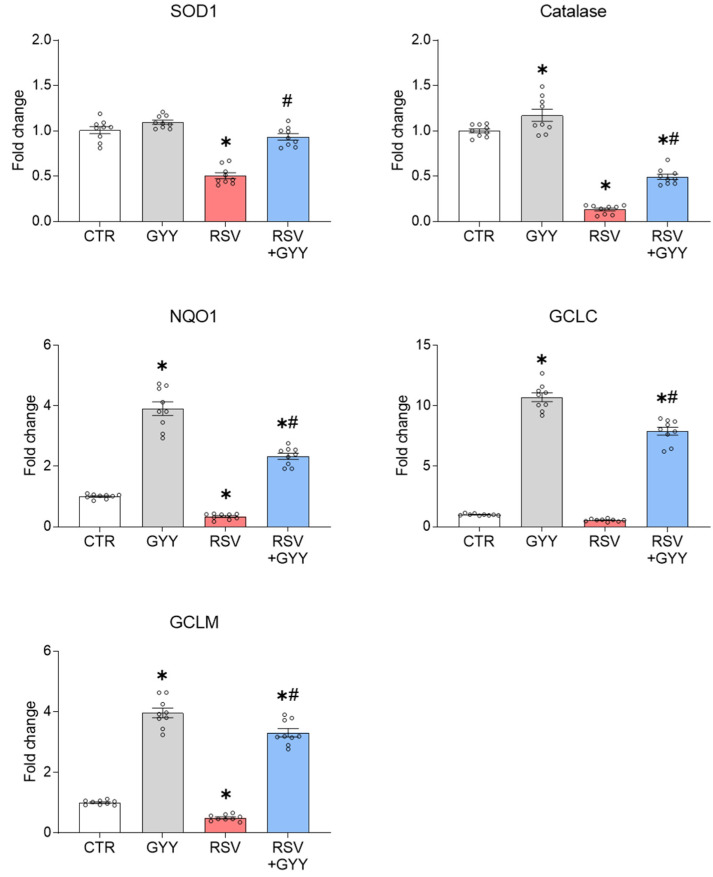
H_2_S donor GYY4137 rescues NRF2-dependent gene expression in RSV infection. SAECs uninfected and infected with RSV were treated with 5 mM GYY4137 and harvested 18 hpi to prepare total RNA. SOD1, catalase, NQO1, GCLC, and GCLM gene expression were quantified by RT-qPCR. Graphs show combined data from three independent experiments expressed as mean ± SEM. The results were analyzed by two-way ANOVA followed by Tukey’s test (* *p* < 0.05 vs. CTR; # *p* < 0.05 vs. RSV).

As a result of the NRF2 degradation, the RSV infection is associated with a significant decrease in the expression and activity of the NRF2 target genes, including AOEs [5]. To determine if GYY4137 could rescue the NRF2-dependent gene expression, the SAECs were infected with RSV, treated with 5 mM GYY4137, and harvested 18 hpi to prepare total RNA. SOD1, catalase, NQO1, GCLC, and GCLM gene expression were analyzed by RT-qPCR. We found that the treatment with GYY4137 led to increased basal levels of catalase, NQO1, GCLC, and GCLM, and significantly upregulated SOD1, catalase, NQO1, GCLC, and GCLM gene expression in SAECs following RSV infection (Figure 2), demonstrating that not only the NRF2 cellular levels are increased by the H_2_S donor, but its function is also restored.

### 3.2. H_2_S Donor GYY4137 Downregulates KEAP1 in RSV Infection

**Figure 3:**
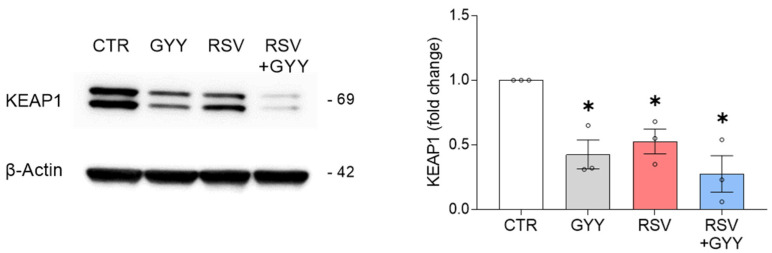
H_2_S donor GYY4137 downregulates KEAP1 in RSV infection. SAECs uninfected and infected with RSV were treated with 5 mM GYY4137 or its vehicle. Cells were harvested 18 hpi and total cell lysates were analyzed by Western blot with anti-KEAP1 antibody. The membrane was reprobed with anti-β-actin antibody for loading control. Western blot image is one representative of three independent experiments. Graph shows the densitometric analysis of KEAP1 after normalization to β-actin expressed as mean ± SEM. Data were analyzed by two-way ANOVA followed by Tukey’s test (* *p* < 0.05 vs. CTR).

The NRF2 expression is tightly regulated [36]. Kelch-like ECH-associated protein 1 (KEAP1) is a regulatory protein that retains NRF2 in the cytoplasm and leads it to ubiquitination and proteasomal degradation. It is known that H_2_S donors can induce KEAP1 sulfhydration, a modification that leads to the NRF2 release, translocation to the nucleus, and promotion of gene transcription [26,37,38,39,40]. To determine whether GYY4137 affected the KEAP1 levels in basal conditions and in the context of RSV infection, the total cell lysates isolated from SAECs infected with RSV and treated with GYY4137 were analyzed by Western blot with anti-KEAP1 antibody. The results showed that both the GYY4137 treatment and RSV infection independently led to a reduction in the KEAP1 protein levels, compared to the control cells. When the RSV-infected cells were treated with GYY4137, we observed a more pronounced reduction in the KEAP1 levels (*p* < 0.01 CTR vs. RSV + GYY), compared to infection alone (*p* < 0.05 CTR vs. RSV) (Figure 3). Although this result suggests that the reduction in KEAP1 protein expression by GYY4137 likely represents an important mechanism responsible for the NRF2 activation in uninfected cells, it would not be sufficient to explain the rescuing of the NRF2 activation and the NRF2-dependent gene expression during RSV infection. This is because we have previously shown that the RSV-induced NRF2 degradation is KEAP1-independent, as it occurs in A549 cells, which carry a KEAP1 mutation, in mouse embryonic fibroblasts (MEFs) derived from KEAP1^−/−^ mice, and in cells lacking KEAP1 by gene silencing [15].

### 3.3. H_2_S Donor GYY4137 Restores NRF2 Ubiquitination and Does Not Affect HDAC Activity in RSV Infection

**Figure 4:**
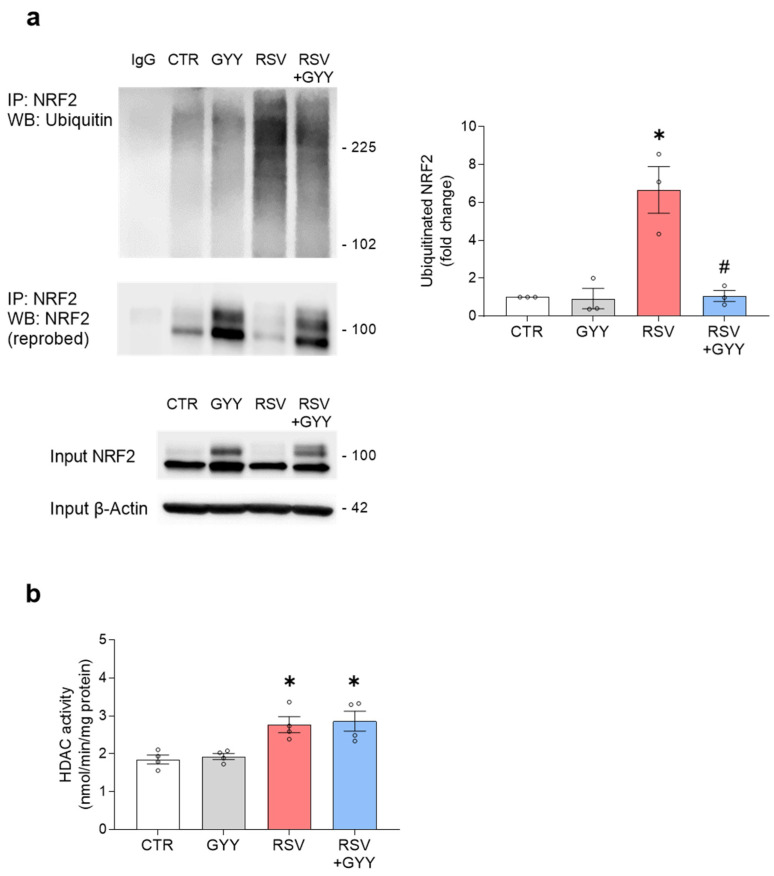
H_2_S donor GYY4137 restores NRF2 ubiquitination and does not affect histone deacetylase (HDAC) activity in RSV infection. (**a**) SAECs uninfected and infected with RSV were treated with 5 mM GYY4137 and harvested 18 hpi. NRF2 was immunoprecipitated from total cell lysates with anti-NRF2 antibody and NRF2 ubiquitination was analyzed by Western blot with anti-ubiquitin antibody. The membrane was stripped and reprobed with anti-NRF2 antibody to determine levels of immunoprecipitated NRF2. A sample of the original pre-immunoprecipitation lysate was also analyzed by Western blot to show levels of NRF2 before immunoprecipitation (input). Western blot images are one representative of three independent experiments. Graph shows densitometric analysis of NRF2 ubiquitination after normalization to immunoprecipitated NRF2 expressed as mean ± SEM. Data were analyzed by two-way ANOVA followed by Tukey’s test (* *p* < 0.05 vs. CTR; # *p* < 0.05 vs. RSV); (**b**) SAECs, uninfected and infected with RSV, were treated with 5 mM GYY4137 and harvested 18 hpi. HDAC activity (class I and class II) in nuclear extracts was measured using HDAC Fluorometric Activity Assay Kit (Cayman Chemical). The HDAC activity was normalized by protein concentration. The graph shows combined data from three independent experiments expressed as mean ± SEM. Data were analyzed by two-way ANOVA followed by Tukey’s test (* *p* < 0.05 vs. CTR).

The NRF2 ubiquitination is an important PTM that targets NRF2 to proteasome degradation. We have previously shown that RSV infection is associated with increased NRF2 ubiquitination, leading to its degradation, as proteasome inhibition rescues the NRF2 cellular levels [17]. To investigate whether the H_2_S donor GYY4137 would affect the NRF2 ubiquitination in the context of RSV infection, NRF2 was immunoprecipitated from whole cell lysates with anti-NRF2 antibody and the NRF2 ubiquitination was detected by Western blot with an anti-ubiquitin antibody. The treatment with the H_2_S donor GYY4137 significantly reduced the RSV-induced NRF2 ubiquitination, with no significant changes in the NRF2 ubiquitination in the absence of infection (Figure 4a). This result indicates that the H_2_S donor GYY4137 affects an important PTM that can determine the fate of NRF2 in the course of RSV infection.

Acetylation is a PTM that regulates the transcriptional activity and DNA binding of NRF2. The acetylation conditions enhance the binding of NRF2 to the ARE, thereby increasing the NRF2-dependent gene transcription. Deacetylation disengages it from the ARE, resulting in transcriptional termination and a decrease in the NRF2-dependent gene transcription [41]. Protein acetylation is regulated by histone acetyltransferases (HATs) and HDACs. The HATs catalyze the transfer of an acetyl group (acetylation), while HDACs catalyze the reverse reaction, removing the acetyl group. Therefore, acetylated proteins are deacetylated by HDACs [42]. We previously showed that RSV infection induces the NRF2 deacetylation and the upregulation of nuclear HDAC activity (class I and class II) [17]. Our previous study indicated that the classes I and II of HDACs are important in regulating the NRF2 function in RSV-infected cells, as the inhibition of their activity was associated with the restoration of the NRF2 nuclear levels and NRF2-dependent gene expression [17]. To determine whether the H_2_S donor treatment would affect the HDAC activity in the context of RSV infection, the nuclear proteins, isolated from the SAECs uninfected and infected with RSV and treated with GYY4137, were used to measure class I and II HDAC activity. Our results showed that the HDAC activity was not significantly affected by the treatment with the H_2_S donor, either in the presence or absence of RSV infection (Figure 4b).

### 3.4. H_2_S Donor GYY4137 Downregulates PML in RSV Infection

**Figure 5:**
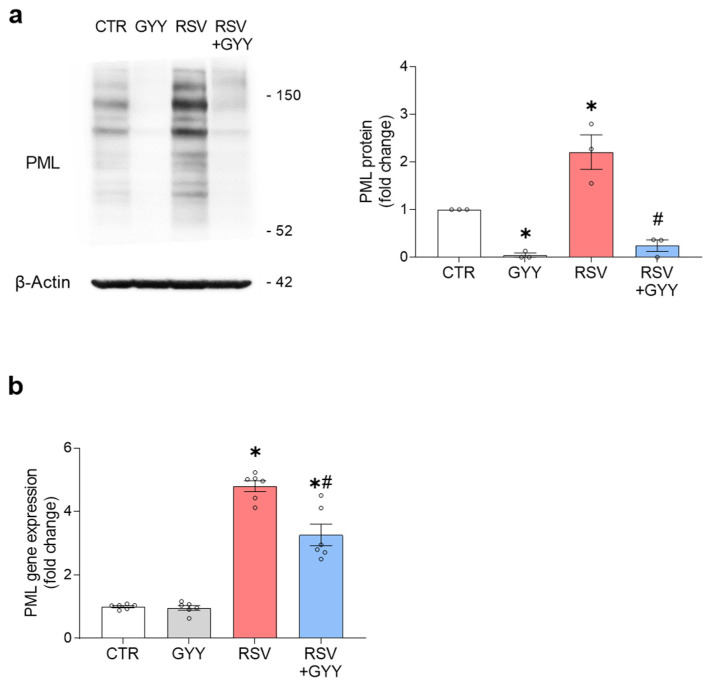
H_2_S donor GYY4137 downregulates PML in RSV infection. (**a**) SAECs uninfected and infected with RSV were treated with 5 mM GYY4137 or its vehicle. Cells were harvested 18 hpi with SDS loading buffer. Whole cell lysates (30 µL) were analyzed by Western blot with anti-PML protein antibody. The membrane was reprobed for anti-β-actin antibody for loading control. Western blot images are one representative of three independent experiments. The graph shows the densitometric analysis of PML after normalization to β-actin expressed as mean ± SEM; (**b**) SAECs uninfected and infected with RSV were treated with 5 mM GYY4137 and harvested 18 hpi to prepare total RNA. PML gene expression was quantified by RT-qPCR. The graph shows combined data from two independent experiments expressed as mean ± SEM. The results were analyzed by two-way ANOVA followed by Tukey’s test (* *p* < 0.05 vs. CTR; # *p* < 0.05 vs. RSV).

In recent investigations we have shown that the RSV-induced NRF2 degradation occurs via the SUMOylation-dependent ubiquitin ligase RING finger protein 4 (RNF4), associated with PML nuclear bodies (PML-NBs). The RSV infection induces the expression of PML protein and PML-NBs formation in an interferon (IFN)-dependent manner [15]. To determine whether H_2_S could modulate PML expression in RSV infection, the total cell lysates from SAECs uninfected and infected with RSV and treated with GYY4137 were analyzed by Western blot, using anti-PML antibody. Surprisingly, the GYY4137 treatment drastically downregulated the PML protein levels, both in uninfected and RSV-infected cells (Figure 5a). We then investigated whether GYY4137 affected PML at the level of gene expression. The uninfected and RSV-infected SAECs were treated with GYY4137 and harvested 18 hpi to prepare the total RNA. The PML gene expression was quantified by RT-qPCR. The SAECs infected with RSV showed a significant upregulation of PML gene expression, which was reduced by the GYY4137 treatment, when compared to infected cells, but still significantly higher than the uninfected control group. The GYY4137 treatment alone did not affect the PML gene expression level in the uninfected cells (Figure 5b).

## 4. Discussion

RSV infection is associated with an imbalance between ROS production and antioxidant defenses, leading to oxidative stress. The transcription factor NRF2 is the master regulator of the expression of the AOEs. In oxidative stress conditions, KEAP1 releases NRF2, which translocates to the nucleus, binds to the ARE sequence, and promotes the gene transcription of its target genes, which include AOEs. However, during RSV infection, NRF2 is degraded through several mechanisms, and the expression and activity of the AOEs are decreased [5,17].

H_2_S, a gaseous molecule that is endogenously produced and has multiple roles, can regulate the cellular antioxidant defenses through the activation of NRF2. Studies have shown that H_2_S donors, H_2_S generating compounds used to demonstrate the effects of H_2_S, can activate the NRF2 antioxidant pathway [43,44,45]. Some studies showed that NaHS can sulfhydrate KEAP1, induce NRF2 dissociation from KEAP1, enhance the NRF2 nuclear translocation, and promote the NRF2-dependent gene expression [26,37]. The effects of H_2_S are complex and depend not only on its concentration, but also on the rate of H_2_S generation [46]. Sulfide salts, such as NaHS and sodium sulfide (Na_2_S), rapidly generate a large burst of H_2_S over a short period of time, which is very different from the slow-release profile of GYY4137, that slowly releases H_2_S over a period of hours [21,47]. The studies specifically using GYY4137 also reported the sulfhydration of KEAP1, activation of NRF2, and induction of genes regulated by NRF2 in experimental models of diabetes-accelerated atherosclerosis, sepsis, HIV-1 latency, renal ischemia/reperfusion injury, and liver damage [32,38,39,40,48]. In our study, we investigated for the first time the effect(s) of the GYY4137 treatment on RSV-induced degradation of NRF2 and inhibition of AOEs. Our results showed that GYY4137 can restore the NRF2 cellular levels, and NRF2-dependent gene expression that were downregulated in RSV infection by multiple distinct mechanism(s).

Our first finding was that the H_2_S donor GYY4137 downregulates the KEAP1 protein levels, an effect even more pronounced during RSV infection, likely leading to increased NRF2 release and activation. This effect alone, however, would not have been sufficient to rescue the NRF2 levels following RSV infection, as we have previously shown that the RSV-induced NRF2 degradation is KEAP1-independent [15].

PTMs, such as ubiquitination and acetylation, are important regulators of transcription factor activation [49]. We previously showed that RSV infection is associated with increased NRF2 ubiquitination and degradation through the proteasome pathway and NRF2 deacetylation [15,17]. In this study, we found that the treatment of RSV-infected cells with GYY4137 dramatically reduces the increase in the NRF2 ubiquitination associated with RSV infection. By reducing the increased NRF2 ubiquitination, the GYY4137 treatment likely suppresses the enhanced NRF2 degradation caused by RSV. The ability of H_2_S to modulate protein ubiquitination was previously reported by Sun et al. [50], who showed that the H_2_S donor NaHS could suppress the levels of myosin heavy chain 6 and myosin light chain 2 ubiquitination in the cardiac tissues of db/db mice. The acetylated proteins are deacetylated by HDACs [42]. The HDACs are subdivided (based on sequence similarities) into four classes [51], and we have previously shown that class I and II HDAC activity is upregulated in RSV infection [17]. In this study, we found that the treatment with the H_2_S donor GYY4137 did not affect the activity of these classes of HDACs, either in uninfected or RSV-infected cells.

Although KEAP1-dependent proteasomal degradation of NRF2 is the most common mechanism, during RSV infection the NRF2 degradation occurs through a different mechanism that involves the PML protein and PML-NBs formation in an IFN-dependent manner. The RSV infection upregulates the PML protein and the inhibition of PML expression results in a reduction in the RSV-associated NRF2 degradation and in the increased AOEs gene expression [15]. Here, we found that the GYY4137 treatment leads to changes in the PML protein levels, both alone and in the context of RSV infection. Although a mild reduction in PML mRNA was observed in infected SAECs, possibly due to a decrease in type I IFN secretion, as we found in GYY4137-treated RSV-infected mice [35], there was no change in the basal PML mRNA levels with the H_2_S donor treatment, suggesting that the decreased PML cellular levels could be due to increased degradation.

In summary, this study shows that the H_2_S donor GYY4137 can activate NRF2 in normal airway epithelial cells and, more importantly, can rescue the NRF2 cellular levels and NRF2-dependent gene expression in the context of RSV infection. In addition to targeting KEAP1, we showed that the GYY4137 treatment affects NRF2 ubiquitination, and the PML protein cellular levels, which regulate the NRF2 stability and degradation following RSV infection.

From our previous studies in vivo, it is evident that NRF2 is a critical regulator of innate, inflammatory, and disease-associated responses in the airways of mice infected with viruses that are members of the *Pneumoviridae* family, as a lack of NRF2 is associated with decreased antiviral response, increased airway neutrophilia, and worse clinical disease and airway obstruction following infection [52]. The results of this study support the concept that the exogenous H_2_S administration could be explored as a candidate for potential therapeutic application, to ameliorate the oxidative stress and lung damage associated with RSV infection, in addition to its anti-inflammatory and antiviral properties that we have previously reported, using in vitro and in vivo models of RSV infection and in vitro models of influenza virus and human metapneumovirus (hMPV) [31,34,35]. The previous observation that the effects of H_2_S could be beneficial to other respiratory viral infections suggests that H_2_S could be also explored in the context of severe acute respiratory syndrome coronavirus 2 (SARS-CoV-2), the causative agent responsible for the COVID-19 pandemic, since it has been shown that the expression of the NRF2-driven genes is suppressed in lung biopsies from COVID-19 patients [53].

## Data Availability

Not applicable.

## Supplementary Materials

The following supporting information can be downloaded at: https://www.mdpi.com/article/10.3390/antiox11071410/s1, Figure S1: Hydrogen sulfide (H_2_S) donor sodium hydrosulfide (NaHS) activates NRF2 in primary human small airway epithelial cells (SAECs).
