# Supplementary material for: Hydrogen Sulfide Donor GYY4137 Rescues NRF2 Activation in Respiratory Syncytial Virus Infection
Source: Antioxidants (Basel). 2022 Jul 21;11(7):1410. doi: 10.3390/antiox11071410 (PMC9311616; doi:10.3390/antiox11071410)
Supplement: Supplementary file 1 [file antioxidants-11-01410-s001.zip › antioxidants-1827576-supplementary.pdf]

antioxidants

Supplementary Materials: Hydrogen Sulfide Donor GYY4137 Rescues NRF2 Activation in Respiratory Syncytial Virus Infection

Aline Haas de Mello [1], Tianshuang Liu [1], Roberto P. Garofalo [1,2] and Antonella Casola [1,2*]

[1] Department of Pediatrics, The University of Texas Medical Branch, Galveston, TX 77555, USA; alhaasde@utmb.edu (A.H.M.); tiliu@utmb.edu (T.L.); rpgarofa@utmb.edu (R.P.G.)
[2] Department of Microbiology and Immunology, The University of Texas Medical Branch, Galveston, TX 77555, USA
* Correspondence: ancasola@utmb.edu

Supplementary Figure S1

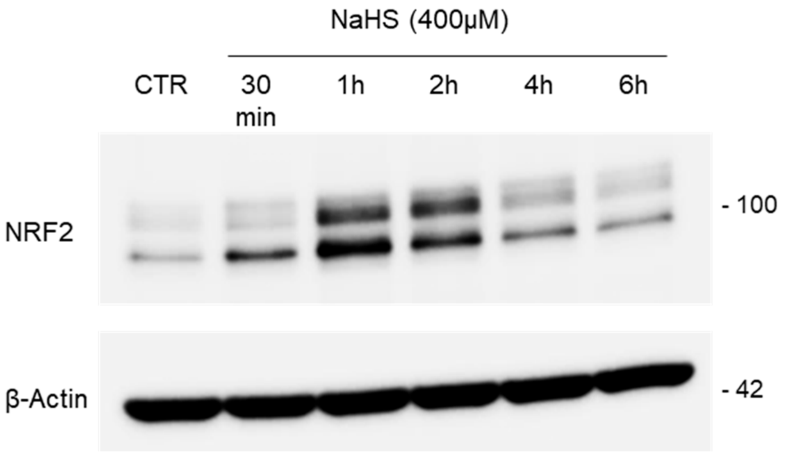

Figure S1. Hydrogen sulfide (H_2S) donor sodium hydrosulfide (NaHS) activates NRF2 in primary human small airway epithelial cells (SAECs). SAECs were treated with 400µM NaHS and harvested at indicated times after treatment. Whole cell lysates were analyzed by western blot with anti-NRF2 antibody. The membrane was reprobed with anti-β-actin antibody for loading control.
